# Gender differences in the progression of target organ damage in patients with increased insulin resistance: the LOD-DIABETES study

**DOI:** 10.1186/s12933-015-0293-1

**Published:** 2015-10-01

**Authors:** Manuel Ángel Gómez-Marcos, José Ignacio Recio-Rodríguez, Leticia Gómez-Sánchez, Cristina Agudo-Conde, Emiliano Rodríguez-Sanchez, JoseAngel Maderuelo-Fernandez, Marta Gomez-Sanchez, Luís García-Ortiz, LOD-DIABETES Group

**Affiliations:** Primary Care Research Unit, The Alamedilla Health Center, Avda. Comuneros 27, 37003 Salamanca, Spain; Castilla and León Health Service–SACYL. REDIAPP, IBSAL, Salamanca, Spain; Medicine Department, University of Salamanca, Salamanca, Spain; LOD-DIABETES Group, redIAPP: Research Network on Preventive Activities and Health Promotion, 37003 Salamanca, Spain

**Keywords:** Insulin resistance, Target organ damage, Gender difference, Drug treatment

## Abstract

**Background:**

The purpose of this study was to analyze the evolution of vascular, cardiac and renal target organ damage (TOD) in patients with increased insulin resistance over a 3.5 year follow-up and to investigate gender difference and factors that influence its progression.

**Methods:**

We performed a prospective observational study involving 112 patients (71 men, 41 women) who were followed for 3.5 years. Measurements included blood pressure, blood glucose, lipids, smoking, body mass index (BMI) and HOMA-Ir Vascular TOD included carotid intima-media thickness (IMT), pulse wave velocity (PWV) and ankle/brachial index (ABI). Cardiac TOD included Cornell voltage-duration product and Sokolow. Renal TOD included creatinine, glomerular filtration and albumin/creatinine ratio.

**Results:**

The IMT increased in both genders. Each year, the IMT increased 0.005 mm in men and 0.011 in women and the PWV 0.024 and 0.020 m/sec, respectively. The highest increase was in women with type 2 diabetes mellitus, who had an increase in TOD carotid (40 %), PWV (24 %) and renal TOD (20 %). Multiple regression analysis, after adjusting for age and gender, showed a negative association between duration since diabetes diagnosis and ABI (β = −0.006; p = 0.017) and between BMI and glomerular filtration (β = −0.813; p = 0.014). HbA1c was positively associated with PWV (β = 0.501; p = 0.014).

**Conclusions:**

This study showed that the progression of vascular and renal TOD differs by gender. The increase in vascular and renal TOD was higher in women, especially in diabetic women. The PWV increase showed a positive association with mean HbA1c levels during the follow-up. Glomerular filtration was associated with BMI and the ABI was associated with duration since type 2 diabetes mellitus diagnosis.

Trial registration: Clinical Trials.gov Identifier NCT01065155

## Background

Cardiovascular disease (CVD) morbidity and mortality is greater in people with increased insulin resistance [(type 2 diabetes mellitus (T2DM) or metabolic syndrome (MetS)] [1–3]. The presence of vascular [4–6], cardiac [7] and renal [8, 9] target organ damage (TOD) increases the risk of cardiovascular complications independent of the existing estimated risk [10]. In the general population the overall median age for evident CVD is about 9 years lower in men than in their women counterparts worldwide [11], except in diabetic women who have a higher risk of CVD than diabetic men [12].

In the general population, left ventricular hypertrophy (LVH) is more prevalent in men [13] and peripheral arterial disease is more prevalent in women [14]. However, carotid intima-media thickness (IMT) and pulse wave velocity (PWV) are higher in men, although this difference is attenuated with age [15]. Measurement of IMT increases the capacity to identify individuals with diabetes [16] or MetS [17] who are at a high risk of developing cardiovascular disease. Measurement of PWV increases the capacity to identify individuals with diabetes [16] or glucose intolerance [18], healthy individuals [19] and subjects with moderate or intermediate risk [20] who are at a high risk of developing cardiovascular disease. Patient age and gender are the most important determinants of IMT [21]. In the general population, the mean estimates of IMT progression ranged from 0.001 to 0.030 mm per year for the common carotid artery IMT, the mean estimates of IMT progression ranged from 0.001 to 0.030 mm per year for common carotid artery IMT, decreasing with control of the risk factors in diabetic individuals [22]. Postprandial blood glucose and HbA1c have a greater influence upon IMT than basal blood glucose [23], but there was no association to cardiovascular events [24]. The predictors of PWV include abdominal obesity, hyperglycemia and dyslipidemia [15]. It has also been reported that adequate glucose and blood pressure control decreases the PWV progression in diabetic patients [25].

In diabetic men, albuminuria occurs more frequently and with a greater reduction in glomerular filtration rate than in diabetic women [26, 27], but LVH is more prevalent in diabetic women than in diabetic men [25].

However, differences in the evolution of the TOD between T2DM and MetS have been little studied [28], and the role that the gender plays in vascular, cardiac and renal TOD in patients with increased insulin resistance, remains unclear and there have been few longitudinal studies.

Therefore, we analyzed the evolution of vascular, cardiac and renal TOD in patients with increased insulin resistance over a 3.5-year follow-up and investigated gender differences and factors that influence TOD progression.

## Methods

### Study design

A prospective observational study was performed in a primary care setting, with a median of follow up of 3.5 years (range, 2.9–3.9 years). This study included 112 subjects from the LOD-DIABETES study (NCT01065155) [29].

### Study population

Using consecutive sampling, we included 112 patients with increased insulin resistance considering clinical criteria, i.e. with diagnosis of T2DM (n = 68) or MetS (n = 44). Type 2 diabetes mellitus (T2DM) defined using the American Diabetes Association criteria [30] or MetS defined according to the National Cholesterol Education Program, ATP III [31]. The study included patients with diabetes or metabolic syndrome, who visited their family physician between January 2009 and January 2010, and these patients included 71 men (63.4 %) and 41 women (36.6 %). Exclusion criteria were as follows: patients who were unable to comply with the protocol requirements (psychological and/or cognitive disorders; failure to cooperate; educational limitations and problems in understanding written language; and failure to sign the informed consent document). Patients participating in a clinical trial during the study were also excluded, as were patients with serious life-threatening comorbidities in the previous 12 months.

The sample size was estimated to detect a statistically significant difference in the carotid IMT that was greater than or equal to 0.03 mm between baseline and the fourth assessment. Accepting an alpha risk of 0.05 and a beta risk of 0.2 in a two-sided test, 112 subjects were necessary to achieve a statistically significant difference. The standard deviation was estimated to be 0.11 mm, and the drop-out rate was estimated as 5 %. An independent ethics committee of Salamanca Health Area (Spain) approved the study. All participants gave written informed consent, according to the general recommendations of the Declaration of Helsinki [32].

### Measurements

A detailed description has been published elsewhere of how clinical data were collected, including anthropometric measurements, blood pressure and TOD assessment [29].

### Blood pressure

Three measurements of systolic (SBP) and diastolic blood pressure (DBP) were collected using a validated OMRON model M7 sphygmomanometer (Omron Health Care, Kyoto, Japan). We used the average of the last two measurements according to the recommendations of the European Society of Hypertension [33]. Mean blood pressure (MBP) was calculated as the sum of SBP + 2 × DBP, divided by 3.

### Vascular assessment

#### Carotid femoral pulse wave velocity

PWV was estimated using the SphygmoCor System (AtCor Medical Pty Ltd., Head Office, West Ryde, Australia), with patients in the supine position. The pulse wave of the carotid and femoral arteries were analyzed to estimate the ECG wave delay and calculate the PWV. Distance measurements from the sternal notch to the carotid and femoral arteries at the sensor location were collected using a measuring tape and multiplied by 0.8, as recommended by expert consensus [34]. TOD was indicated if the PWV was greater than 10 m/sec [33].

#### Carotid intima media thickness

Carotid ultrasound to assess carotid IMT was performed by two investigators trained for this purpose before starting the study. A Sonosite Micromax ultrasound (Sonosite Inc., Bothell, Washington, USA) device paired with a 5–10 MHz multi-frequency high-resolution linear transducer with Sonocal software was used for automatic measurement of IMT to optimize reproducibility. Common carotid artery measurements were made after examining a 10-mm longitudinal section 1 cm from the bifurcation. Measurements were performed at the proximal and distal wall in the lateral, anterior and posterior projections. They followed an axis perpendicular to the artery to discriminate between two lines: one for the intima-blood interface and the other for the media-adventitious interface. A total of 6 measurements were obtained for the right carotid and 6 measurements for the left carotid. We used the average values (average IMT) that were automatically calculated using the software [35]. The measurements were obtained with the subject lying down, with their head extended and slightly turned opposite of the carotid artery under study. Average IMT values were considered abnormal if they were >0.90 mm, if there were atherosclerotic plaques with a diameter of 1.5 mm or if there was a focal increase of 0.5 mm or 50 % of the adjacent IMT [33].

### Evaluation of peripheral artery involvement

Peripheral artery involvement was assessed using the ankle-brachial index (ABI) and was calculated in the morning for patients who had not drank coffee or smoked tobacco for at least 8 h prior to the measurement. The room temperature was 22–24 °C. Patients were supine with the feet uncovered. The pressure in the lower limbs was measured after resting for 20 min using a portable Watch BP Office for assessing the ABI (Microlife AG Swiss Corporation Espenstrasse 139; CH-9443 Widnau/Switzerland). The ABI was calculated automatically for each foot by dividing the higher of the two systolic pressures in the ankle by the higher of the two systolic pressures in the arm. An ABI < 0.9 was considered abnormal [33].

### Renal assessment

Kidney damage was assessed by measuring creatinine plasma concentration and glomerular filtration rate (eGFR) as estimated according to the Modification of Diet in Renal Disease-Isotopic Dilution Mass Spectrometry (MDRD-IDMS) [36]. Proteinuria was assessed by the albumin/creatinine ratio, (performing a measurement every year), following the 2013 European Society of Hypertension/European Society of Cardiology Guidelines criteria. TOD was defined as a plasma creatinine of 1.3 mg per 100 ml or higher in men and 1.2 mg per 100 ml or higher in women and an eGFR below 60 ml per min or albumin/creatinine ratio ≥30 mg/g [33].

### Cardiac assessment

The electrocardiographic examination was performed using a General Electric MAC 3.500 ECG System (General Electric, Niskayuna, NY, USA) that automatically measures the voltage and duration of waves and estimates the criteria of the Cornell voltage–duration product (Cornell VDP) and Sokolow Lyon product [37]. The TOD was defined according to the 2013 European Society of Hypertension/European Society of Cardiology Guidelines criteria [33].

### Laboratory determinations

Venous blood sampling was performed between 08:00 and 09:00 after the individuals had fasted and abstained from smoking and the consumption of alcohol and caffeinated beverages for the previous 12 h. Fasting plasma glucose, HbA1c, homeostasis model assessment-insulin resistance (HOMA-IR), creatinine, total cholesterol, triglycerides and high-density lipoprotein (HDL) cholesterol concentrations were measured using standard enzymatic automated methods. Low-density lipoprotein (LDL) cholesterol was estimated using the Friedewald equation. The atherogenic index was estimated by total cholesterol/HDL cholesterol.

The average annual increase in TOD was estimated by dividing the difference between the final and baseline evaluations by the years of follow-up. The individuals performing the various tests were blinded to the clinical data. All assessments were made within 10 days.

#### Statistics

Continuous variables were expressed as the mean ± standard deviation for normally distributed continuous data, the median (interquartile range, IQR) for asymmetrically distributed continuous data and the frequency distribution for categorical data. Statistical normality was tested using the Kolmogorov–Smirnov test. The Student’s *t* test was used to analyze the difference between quantitative variables by gender. The evolution of quantitative variables was analyzed using an analysis of variance for repeated measures, corrected by the Bonferroni method. The presence or absence of sphericity was taken into account and the Greenhouse and Geisser correction was performed.

We used the McNemar and Cochran tests to contrast the hypothesis of two or more related proportions. In addition, we have analyzed the differences in the yearly increase in intima-media thickness (IMT), PWV and estimated glomerular filtration rate (eGFR) in the group of diabetic individuals according to gender. We performed a multiple linear regression analysis, which was adjusted for age and gender, using the stepwise method, one for each of the dependent variable and differences between the fourth measure and baseline (IMT, ABI, cf-PWV, eGFR, Cornell VDP). The independent variables included were: years since diagnosis of diabetes, smoker and mean values taken at the four visits during the 3.5 years of follow-up (atherogenic index, mean blood pressure, body mass index, HbA1C, HOMA-IR, mean number of antihypertensive, lipid lowering and antidiabetic drugs). Data were analyzed using the SPSS version 20.0 statistical package (SPSS Inc., Chicago, Illinois, USA). A value of p < 0.05 was considered statistically significant.

## Results

**Fig. 1:**
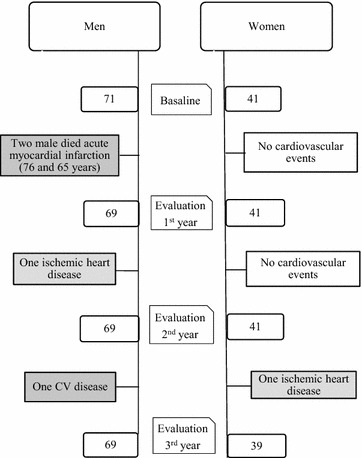
Study flow chart. The subjects analyzed each year are presented in the flow chart. Additional information includes cardiovascular events in each group and evaluation of patients by gender

In the first year of follow-up, two men died as result of acute myocardial infarction. Subsequently, two non-fatal cardiovascular events occurred in the male group. In the female group, there was a non-fatal cardiovascular event. The flow chart is shown in Fig. 1.

The median time from diagnosis of type 2 diabetes mellitus was 9.20 ± 4.16 years. At baseline, the 60.9 % of men and 61.0 % of women had diabetes; 39.1 and 39.0 % had metabolic syndrome; 79.7 and 90.2 % had hypertension; 78.3 and 75.6 % had dyslipidemia and 55.1 and 53.7 % were obese. The mean age was 61 ± 11 years in men and 62 ± 12 in women. We found no statistical differences for gender in any risk factors evaluated.

Table 1 shows the cardiovascular risk factors, biochemical data and drugs analyzed at each of the four evaluations by gender. Table 2 shows the basal and the annual assessments of vascular, cardiac and renal TOD by gender. In men, we observed an increase in IMT in the fourth assessment. In woman, we observed an increase in IMT and in the number of plaques.

**Table 1:** Changes in cardiovascular risk factors and medications used by gender

	Baseline (n=71)	1st year (n=69)	2nd y (n=69)	3rd y (n=69)	p
Men					
Smoker’s, n (%)^†‡§||^	20 (29)	20 (29)	14 (20)	14 (20)	<0.001
Ischemic heart disease n (%)	8 (11)	8 (12)	9 (13)	9 (13)	1.00
Cerebrovascular disease n (%)	2 (3)	2 (3)	2 (3)	3 (4)	0.392
BMI (kg/m^2^)	30.0 ± 4.1	30.3 ± 4.2	30.4 ± 4.4	30.3 ± 4.5	0.526
Total cholesterol (mg/dL)^‡^	198.3 ± 39.3	191.5 ± 41.6	188.9 ± 36.7	183.4 ± 34.4	<0.001
Tryglicerides (mg/dL)	160.6 ± 65.6	153.9 ± 92.8	153.5 ± 101.2	151.7 ± 129.2	0.864
LDL cholesterol (mg/dL)^‡^	121.5 ± 36.2	114.6 ± 34.9	110.6 ± 34.8	109.2 ± 32.9	0.026
HDL cholesterol (mg/dL)^†‡§||^	44.6 ± 9.2	45.4 ± 10.0	48.1 ± 11.4	48.3 ± 11.7	<0.001
Atherogenic index^†‡||^	4.6 ± 1.3	4.4 ± 1.5	4.2 ± 1.3	4.0 ± 1.2	<0.001
Serum glucose (mg/dL)	111.4 ± 32.3	115.3 ± 43.8	112.1 ± 40.7	114.1 ± 37.4	0.759
HbA1c (%)	6.36 ± 1.23	6.51 ± 1.34	6.51 ± 1.06	6.46 ± 1.08	0.493
HOMA-IR	3.2 ± 2.3	4.3 ± 10.5	2.2 ± 1.9	3.0 ± 2.2	0.284
Office SBP (mm Hg)*^‡¶^	141 ± 11	134 ± 15	138 ± 16	132 ± 16	<0.001
Office DBP (mm Hg)*^†‡||¶^	86 ± 10	81 ± 9	82 ± 10	77 ± 9	<0.001
Mean blood pressure (mmHg)^‡||¶^	101 ± 11	99 ± 10	101 ± 10	96 ± 10	<0.001
Mean Antihypertensive drugs*^†‡^	1.07 ± 1.06	1.49 ± 1.26	1.61 ± 1.32	1.64 ± 1.31	<0.001
Antihypertensive drugs n (%)^†‡^	42 (60.9)	50 (72.5)	51 (73.9)	52 (75.4)	<0.001
Mean Lipid lowering drugs^†‡^	0.54 ± 0.53	0.62 ± 0.55	0.68 ± 0.61	0.68 ± 0.58	<0.001
Lipid lowering drugs n (%)^†‡^	36 (52.2)	41 (59.4)	42 (60.9)	43 (62.3)	0.048
Mean antidiabetic drugs^†‡^	0.75 ± 0.81	0.83 ± 0.82	0.91 ± 0.92	0.86 ± 0.88	0.048
Antidiabetic drugs, n (%)	38 (55.1)	42 (60.9)	42 (60.9)	42 (60.9)	0.875

	Baseline (n = 41)	1st year (n = 41)	2nd year (n = 41)	3rd year (n = 41)	p
Women					
Smoker’s, n (%)	5 (12.2)	5 (12.2)	5 (12.2)	5 (12.2)	1.00
Ischemic heart disease, n (%)	2(5)	2 (5)	3 (7)	3 (7)	0.392
Cerebrovascular disease, n (%)	0	0	0	0	–
BMI (kg/m^2^)	30.0 ± 4.1	30.3 ± 4.2	30.4 ± 4.4	30.3 ± 4.5	0.526
Total cholesterol (mg/dL)	204.1 ± 43.8	201.2 ± 37.4	196.2 ± 26.8	188.4 ± 27.2	0.099
Tryglicerides (mg/dL)	140.8 ± 58.7	129.9 ± 40.5	138.4 ± 61.1	129.6 ± 50.5	0.671
LDL cholesterol (mg/dL)	121.9 ± 37.5	121.8 ± 31.1	112.8 ± 25.6	109.9 ± 24.0	0.084
HDL cholesterol (mg/dL)^†§^	51.9 ± 13.6	53.5 ± 13.6	55.8 ± 14.1	53.3 ± 11.4	0.027
Atherogenic index	4.1 ± 1.1	3.9 ± 1.0	3.7 ± 1.1	3.7 ± 1.1	0.069
Serum glucose (mg/dL)	116.7 ± 34.7	114.5 ± 39.3	117.4 ± 40.4	110.6 ± 28.9	0.606
HbA1c (%)	6.37 ± 1.11	6.39 ± 1.04	6.53 ± 1.04	6.51 ± 1.04	0.220
HOMA-IR	3.1 ± 2.5	4.2 ± 11.6	2.9 ± 5.0	2.6 ± 2.2	0.175
Office SBP (mm Hg)	134 ± 17	133 ± 21	131 ± 15	131 ± 17	0.738
Office DBP (mm Hg)^‡^	83 ± 12	81 ± 12	78 ± 10	77 ± 10	<0.001
Mean Blood Pressure (mmHg)	97 ± 10	98 ± 13	97 ± 10	95 ± 11	0.260
Mean Antihypertensive Drugs	1.46 ± 1.27	1.54 ± 1.12	1.78 ± 1.17	1.66 ± 0.94	0.133
Antihypertensive Drugs n (%)^†^	29(70.7)	32 (78.0)	34 (82.9)	36 (87.8)	<0.001
Mean Lipid lowering drugs^‡||^	0.56 ± 0.59	0.56 ± 0.59	0.68 ± 0.57	0.71 ± 0.56	0.017
Lipid lowering drugs, n (%)^†‡^	21 (51.2)	22 (53.7)	26 (63.4)	27 (65.9)	0.033
Mean antidiabetic drugs^†^	0.95 ± 0.97	0.85 ± 0.96	1.04 ± 1.07	0.93 ± 0.96	0.108
Antidiabetic drugs, n (%)	24 (60.9)	24 (60.9)	24 (60.9)	24 (60.9)	1.000

Data for qualitative variables are expressed as n: number (%) and quantitative variables as mean ± standard deviation

*BMI* body mass index, *LDL* low density lipoprotein, *HDL* high density lipoprotein, *HbA1C* glycosylated hemoglobin, *HOMA-IR* homeostasis model assessment insulin resistance, *SBP* systolic blood pressure, *DBP* diastolic blood pressure

p < 0.05: between * baseline and 1st year; ^†^baseline and 2nd year; ^‡^baseline and 3rd year

p < 0.05: between ^§^1st and 2nd year; ^||^1st and 3rd year

p < 0.05 between ^¶^2nd and 3rd year

**Table 2:** Changes in target organ damage and arterial stiffness by gender

Men	Baseline (n = 71)	1st year (n = 69)	2nd year (n = 69)	3rd year (n = 69)	p
Vascular					
C-IMT average (mm)^¶^	0.779 ± 0.127	0.776 ± 0.123	0.769 ± 0.123	0.796 ± 0.126	0.005
Plaques carotid n (%)	16 (23.2)	15 (21.7)	19 (27.5)	21 (30.4)	0.148
ABI*	1.15 ± 0.11	1.19 ± 0.12	1.18 ± 0.13	1.16 ± 0.11	<0.001
PWV (m/s)	9.31 ± 2.32	9.33 ± 2.64	9.68 ± 2.37	9.48 ± 2.47	0.661
Renal					
Serum creatinine (mg/dL)	0.95 ± 0.14	0.97 ± 0.18	0.93 ± 0.17	0.96 ± 0.18	0.110
eGFR (mL/min/1.73 m^2^)	89.5 ± 16.8	88.5 ± 18.3	92.3 ± 19.8	87.9 ± 18.2	0.072
Albumin/creatinine ratio (mg/g)	34.8 ± 81.4	44.0 ± 122.9	37.7 ± 113.8	45.9 ± 156.8	0.826
Heart					
Cornell VDP (mm/ms)	1487 ± 573	1538 ± 506	1515 ± 543	1458 ± 535	0.285
Sokolow (mm/ms)*^§||^	20.3 ± 5.9	18.7 ± 6.2	21.3 ± 6.6	20.4 ± 6.8	<0.001

Women	Baseline (n = 41)	1st year (n = 41)	2nd year (n = 41)	3rd year (n = 41)	p
Vascular					
C-MT average (mm)^§^	0.715 ± 0.092	0.728 ± 0.076	0.726 ± 0.099	0.754 ± 0.115	0.034
Plaques carotid, n (%)^†§^	3 (7.3)	3 (7.3)	6 (14.6)	11 (26.8)	<0.001
ABI*^†‡^	1.07 ± 0.10	1.17 ± 0.09	1.18 ± 0.10	1.14 ± 0.09	<0.001
PWV (m/s)	9.74 ± 2.70	9.21 ± 2.11	9.14 ± 2.34	9.79 ± 2.92	0.286
Renal					
Serum creatinine (mg/dL)	0.73 ± 0.11	0.73 ± 0.12	0.70 ± 0.13	0.72 ± 0.14	0.285
eGFR (mL/min/1.73 m^2^)	89.1 ± 16.6	93.2 ± 22.6	95.0 ± 16.6	93.1 ± 22.1	0.163
Albumin/creatinine ratio (mg/g)	14.6 ± 37.4	6.6 ± 6.6	3.0 ± 5.6	9.1 ± 14.8	0.122
Heart					
Cornell VDP (mm/ms)	1741 ± 598	1756 ± 542	1852 ± 680	1838 ± 740	0.430
Sokolow (mm/ms)^§^	19.1 ± 6.1	19.4 ± 5.3	20.5 ± 5.6	19.6 ± 5.7	0.013

Data for qualitative variables are expressed as n: number and (%) and quantitative variables as mean ± standard deviation

*C* Carotid, *IMT* intima media thickness carotid, *ABI* ankle brachial index, *PWV* pulse wave velocity, *eGFR* estimated glomerular filtration rate, *Cornell VDP* cornell voltage duration product

p < 0.05: between * baseline and 1st year; ^†^baseline and 2nd year; ^‡^baseline and 3rd year

p < 0.05: between ^§^1st and 2nd year; ^||^1st and 3rd year

p < 0.05 between ^¶^2nd and 3rd year

Each year in men and women, the IMT increased 0.005 mm and 0.011, the PWV increased 0.024 and 0.020 m/sec and the eGFR increased -2.35 and 4.02 mL/min/1.73 m, respectively. The highest increase was in women with type 2 diabetes mellitus (Table 3).

**Table 3:** Changes in target organ damage by gender

	Global	Men	Women	p
	n = 112	n = 61	n = 41	
Vascular				
C IMT average baseline (mm)	0.775 ± 0.121	0.779 ± 0.127	0.715 ± 0.092	0.003
C IMT average final (mm)	0.780 ± 0.123	0.796 ± 0.126	0.754 ± 0.115	0.084
Difference (final–baseline)	0.025 ± 0.077	0.017 ± 0.070	0.039 ± 0.087	0.147
Annual increase	0.007 ± 0.023	0.005 ± 0.021	0.011 ± 0.025	0.215
Annual increase in diabetic patients	0.011 ± 0.002	0.007 ± 0.022	0.018 ± 0.027	0.021
ABI baseline	1.129 ± 0.121	1.116 ± 0.120	1.080 ± 0.109	0.001
ABI final	1.179 ± 0.107	1.190 ± 0.11	1.160 ± 0.09	0.128
Difference (final–baseline)	0.049 ± 0.148	0.033 ± 0.155	0.076 ± 0.135	0.142
Annual increase	0.015 ± 0.044	0.010 ± 0.045	0.022 ± 0.021	0.157
Annual increase in diabetic patients	0.011 ± 0.042	0.004 ± 0.671	0.021 ± 0.512	0.157
PWV baseline (m/sec)	9.470 ± 2.467	9.310 ± 2.324	9.740 ± 2.695	0.393
PWV final (m/sec)	9.594 ± 2.643	9.476 ± 2.472	9.788 ± 2.922	0.507
Difference (final–baseline)	0.108 ± 2.255	0.146 ± 2.029	0.046 ± 2.603	0.551
Annual increase	0.022 ± 0.672	0.020 ± 0.58	0.024 ± 0.80	0.835
Annual increase in diabetic patients	0.178 ± 0.632	0.098 ± 0.512	0.302 ± 0.780	0.003
Renal				
eGFR baseline (mL/min/1.73 m^2^)	89.383 ± 16.658	89.542 ± 16.810	89.115 ± 18.190	0.897
eGFR final (mL/min/1.73 m^2^)	89.898 ± 18.834	87.891 ± 18.19	93.129 ± 22.07	0.207
Difference (final–baseline)	−1.341 ± 20.100	−2.349 ± 21.253	4.015 ± 16.917	0.022
Annual increase	−0.170 ± 4.945	−0.732 ± 4.698	1.184 ± 5.218	0.104
Annual increase in diabetic patients	−0.308 ± 5.095	−1.286 ± 5.301	1.257 ± 4.406	0.052
Heart				
Cornell VDP baseline (mm/ms)	1604 ± 645	1487 ± 573	1741 ± 598	0.032
Cornell VDP final (mm/ms)	1568 ± 594	1458 ± 535	1838 ± 740	0.006
Difference (final–baseline)	36 ± 371	−31 ± 291	97 ± 471	0.229
Annual increase	12 ± 111	−9 ± 29	31 ± 141	0.206
Annual increase in diabetic patients	6 ± 101	−8 ± 76	29 ± 131	0.430

Data for qualitative variables are expressed as n: number and (%) and quantitative variables as mean ± standard deviation

*Y* year, *C* carotid, *IMT* intima media thickness carotid, *ABI* ankle brachial index, *PWV* pulse wave velocity, *eGFR* estimated glomerular filtration rate, *Cornell VDP* cornell voltage duration product, *p* gender differences

**Fig. 2:**
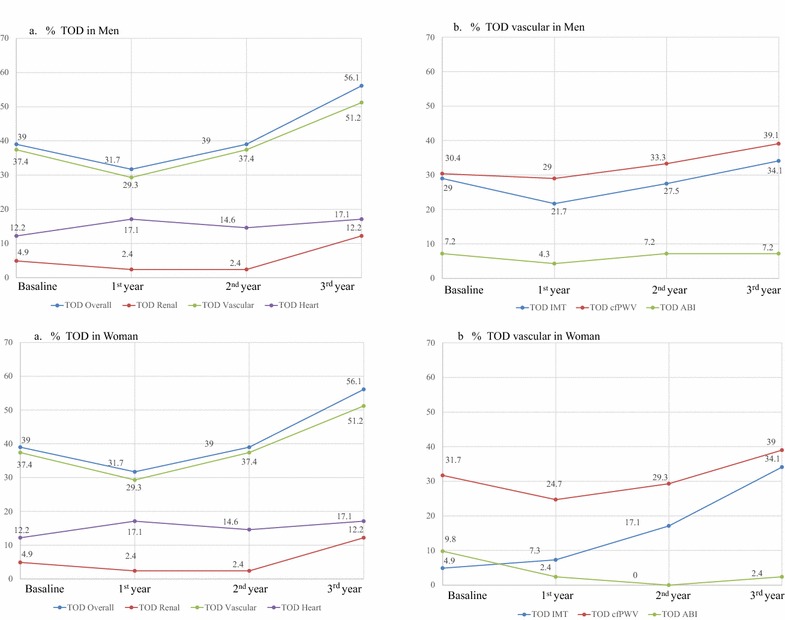
Changes in target organ damage over time during 3.5 years of follow-up. **a** Target organ damage (TOD) in men and in women; **b** Vascular TOD in men and in women. *IMT* intima media thickness, *cf-PWV* carotid femoral pulse wave velocity, *ABI* ankle brachial index. In men, the vascular TOD was significantly different between the final and baseline assessment and the 2nd and 3rd assessments (p < 0.05). The cf-PWV TOD was also significantly different between the final and baseline assessment (p < 0.05). In women, the vascular TOD was significantly different between the 2nd and 3rd assessments (p < 0.05). There was also a significant difference in the IMT TOD between the baseline and 2nd assessment and between the 2nd and 3rd assessment (p < 0.05). The cf-PWV TOD was significantly different between the 2nd and 3rd assessment (p < 0.05)

Figure 2 shows the trend and the percentage of participants, by gender, who had vascular, cardiac and renal TOD at each of the four time points. The overall TOD increased in men and women by 10 and 17 %, respectively. In men and women, the overall vascular TOD increased by 11.5 and 14 %, the overall renal TOD increased by −8 and 7 %, and the overall cardiac TOD increased by 1.5 and 5 %, respectively.

Multiple regression analysis, using the Stepwise method, was performed after adjusting for age and gender, and it showed a negative association between the duration since diagnosis of diabetes and ABI changes during the follow up (β = −0.006; p = 0.017), and between the BMI and glomerular filtration changes (β = −0.813; p = 0.014). The HbA1c was positivenly associated with PWV changes (β = 0.501; p = 0.014; Table 4).

**Table 4:** With multiple regression analysis evolution of TOD and vascular structure and function parameters as dependent variables and average risk factors and drugs used as independent variable

Dependent variable	β	CI 95 %	p value
IMT average mean			
Age	0.001	−0.001 to 0.002	0.651
Sex	0.022	−0.009 to 0.052	0.158
ABI			
Age	0.003	0.001 to 0.0060	*0.028*
Sex	0.048	−0.010 to 0.105	0.104
Years of evolution of diabetes	−0.006	−0.011 to −0.001	*0.017*
PWV			
Age	0.044	0.007 to 0.081	*0.021*
Sex	0.111	−0.958 to 0.736	0.796
HbA1C-mean	0.501	0.102 to 0.901	*0.014*
eGFR			
Age	−0.222	−0.493 to −0.049	0.107
Sex	5.557	−0.630 to 11.744	0.078
BMI-mean	−0.813	−1.461 to −0.166	*0.014*
Cornell VDP			
Age	2.417	−3.990 to 8.824	0.456
Sex	9.572	−5.091 to 24.235	0.198

Dependent variable: Differences between the final and baseline assessment: (*IMT* intima-media thickness of common carotid artery, *ABI* ankle brachial index, *PWV* pulse wave velocity, *eGFR* estimated glomerular filtration rate, *Cornell VDP* cornell voltage duration product)

Muliple linear regression by Stepwise method adjusted for: Age and gender (1 = men, 2 = women)

Indepedent variable: years of evolution of diabetes. Smoker’s (1 = Yes, 0 = No). Mean values of the four measurements taken during the follow-up: (atherogenic index. Mean blood pressure. Body mass index. HbA1c; HOMA-IR mean: (Homeostasis Model Assessment Insulin Resistance). Antihypertensive drugs mean. Lipid lowering drugs mean and antidiabetic drugs mean)

Italic values indicate statistical association

## Discussion

This study included a 3.5-year median follow-up period of patients with increased insulin resistance, and it showed five important findings: (1) increased carotid TOD was higher in women; (2) increased renal TOD was higher in women and eGFR had a negative association with BMI; (3) the largest increase in IMT, PWV and renal TOD was in the group of diabetic women; (4) poor glycemic control involves greater progression of PWV; and (5) the ABI had a negative association with the duration since diagnosis of diabetes.

We found that IMT progressed 0.005 mm annually in men, 0.011 mm in women and 0.018 mm in diabetic women. Our results are consistent with those published by Zhao et al. [38] in diabetic women (0.022 mm/year), but lower than the increase IMT observed in men (0.030 mm/year). This could be because of differences in age, ethnicity, BMI and the number of comorbidities. As in other studies with the general population [24] and diabetics [38], we have not found any variable that explains the progression of IMT in patients with insulin resistance. The increase in IMT and the number of plaques during the follow-up period was higher in females with type 2 diabetes, supporting the results of previous studies that showed that women with diabetes have a risk of cardiovascular events that is 2-times greater than that in men [12, 39].

The global annual progression of PWV in our study was 0.020 m/sec in men, 0.024 m/sec in women and 0.302 m/sec in diabetic women. Our results are similar to those published by Marcel et al. [25] in type 2 diabetic patients (the annual increase of PWV was 0.11 m/sec) and support the data reported by De Angelis et al. [40], which suggests that arterial stiffness and its progression is greater in women with type 2 diabetes mellitus. Similar to previous study results [25], our multivariate linear regression analysis showed a direct association between changes in PWV and mean HbA1c during follow-up. These findings have important clinical implications because the improvement in glycemic control could reduce aortic stiffness, thus reducing the burden of morbidity and mortality associated with type 2 diabetes.

We also found a negative association between age, BMI and eGFR, which is in line with previously-published results in patients with type 2 diabetes mellitus [26, 41]. The trend for the ABI was similar in both sexes, for the absolute values and the percentage of patients with ABI < 0.9.

The evaluation of left ventricular hypertrophy was based on the Sokolow criteria and it showed differences between the four measurements in both sexes. However, the results did not show a clear trend, and there was no variable explaining these changes [13].

In summary, our results were similar to previously-published results [12, 42], indicating that women with type 2 diabetes are more vulnerable than men to develop vascular and renal TOD in the pre-occurrence phases of cardiovascular disease.

The poorer evolution of vascular and renal TOD in females, particularly among those with diabetes, may be due to the fact that women show greater stiffness values in the prepubertal period, and this stiffness moreover increases after menopause. This suggests that women intrinsically have stiffer large arteries than men—though these effects are mitigated by the sexual steroids during the reproductive years [43, 44]. Other factors that can influence the gender differences are: the sexual dimorphism of lipid metabolism, these differences include how dietary fatty acids are handled, the secretion and clearance of very low-density lipoprotein-triglycerides, the release rates of free fatty acids from adipose tissue relative to energy needs, and the removal of free fatty acids from the circulation, including the storage of free fatty acids into adipose tissue via the direct uptake process [45, 46] and inflammatory factors [47].

The results of this study will help medical professionals in their routine practice to consider gender differences, particularly as regards the group of diabetic women, with a view to performing better evaluation of vascular, renal and cardiac TOD, and thus affording better treatment suited to each individual case.

This study has some limitations that must be considered. First, the small number of subjects per group limits the power of analysis. Second, creatine albumin index was determined in a single sample each year. Also, at the time of viewing these results, the subjects included in the study had multiple associated pathologies and were being treated with many drugs, which may have affected the TOD values. It is also limited to 3.5 years of follow-up. In addition, these patients were not randomized but were involved consecutive sampling.

In conclusion, this study showed that the progression of vascular and renal TOD differs by gender. The increase of vascular and renal TOD was higher in women, especially in diabetic women. The increase in PWV showed a positive association with mean HbA1c in the follow-up period. Glomerular filtration was associated with BMI and the ABI was associated with duration since type 2 diabetes mellitus diagnosis.

## Abbreviations

ABI: ankle-brachial index
BMI: body mass index
BP: blood pressure
cf-PWV: carotid femoral pulse wave velocity
CVD: cardiovascular disease
Cornell VDP: cornell voltage-duration product
DBP: diastolic blood pressure
MDRD-IDMS: modification of diet in renal disease-isotopic dilution mass spectrometry
eGFR: glomerular filtration rate
IMT: ntima-media thickness
LVH: left ventricular hypertrophy
PWV: pulse wave velocity
TOD: target organ damage
SBP: systolic blood pressure
T2DM: type 2 diabetes mellitus
